# Transmission Dynamics and Final Epidemic Size of Ebola Virus Disease Outbreaks with Varying Interventions

**DOI:** 10.1371/journal.pone.0131398

**Published:** 2015-07-21

**Authors:** Maria Vittoria Barbarossa, Attila Dénes, Gábor Kiss, Yukihiko Nakata, Gergely Röst, Zsolt Vizi

**Affiliations:** 1 Bolyai Institute, University of Szeged, Szeged, Hungary; 2 Graduate School of Mathematical Sciences, University of Tokyo, Tokyo, Japan; China Medical University, TAIWAN

## Abstract

The 2014 Ebola Virus Disease (EVD) outbreak in West Africa was the largest and longest ever reported since the first identification of this disease. We propose a compartmental model for EVD dynamics, including virus transmission in the community, at hospitals, and at funerals. Using time-dependent parameters, we incorporate the increasing intensity of intervention efforts. Fitting the system to the early phase of the 2014 West Africa Ebola outbreak, we estimate the basic reproduction number as 1.44. We derive a final size relation which allows us to forecast the total number of cases during the outbreak when effective interventions are in place. Our model predictions show that, as long as cases are reported in any country, intervention strategies cannot be dismissed. Since the main driver in the current slowdown of the epidemic is not the depletion of susceptibles, future waves of infection might be possible, if control measures or population behavior are relaxed.

## Introduction

Ebola virus was originally named *Zaire Ebola Virus* after the country Zaire (now the Democratic Republic of Congo), where it first appeared in 1976 [1, 2]. Nowadays five Ebola virus strains have been identified, four of which cause severe hemorrhagic fever in humans [3, 4]. Fruit bats have been confirmed as natural Ebola virus hosts. The virus is transmitted to humans through close contact with blood, secretions, organs or other bodily fluids of infected ill or dead animals. Human-to-human transmission follows through direct contact with blood, secretions, organs or other bodily fluids of infected people, as well as with materials (e. g., bedding, clothing) contaminated with these fluids [4]. Ebola virus may be transmitted even from dead patients, indeed these remain infectious as long as their blood and body fluids contain the virus.

After an incubation period which varies from 2 to 21 days, the patient shows the first symptoms, such as fever, fatigue, muscular pains, headache and sore throat. The illness becomes more acute with vomiting, diarrhea, body rash, tremors and in some cases, both internal and external bleeding. Ebola Virus Disease (EVD) is fatal, with a case fatality around 50–70% [5].

The 2014 EVD outbreak in West Africa was the largest and longest ever reported since the first identification of this disease [6]. Retrospective studies by the World Health Organization (WHO) identified an 18-month-old boy, fallen ill in Guinea on 26 December 2013, as the first Ebola victim. Since then, the virus spread and caused almost 30 deaths in Guinea, before being identified as Ebola at the end of March 2014. The virus rapidly spread to Liberia (first case reported on 30 March) and Sierra Leone (first case reported on 26 May) causing more and more deaths. It was further imported via air travel into Nigeria, Mali, Senegal, Spain, the United Kingdom and the United States of America, causing minor outbreaks [6]. As of 25 January 2015, a total of 22096 EVD cases and 8810 deaths were reported [7]. Out of these, only 35 cases and 15 deaths occurred outside of the most affected countries, Guinea, Liberia and Sierra Leone [7].

After a continuous increase in the case incidence, weekly case numbers started to decrease in Liberia (from mid September), in Sierra Leone (from mid December) and finally in Guinea (from the beginning of January 2015) [7]. The current situation in West Africa allows to hope for the end of Ebola epidemic. Nevertheless, as long as cases are reported in any country, the introduction of an infectious patient into unaffected regions remains a danger.

In 2014, huge efforts were made by international organizations to keep the West African epidemic under control and avoid disease spread. Mathematical modeling has contributed to the investigation of the disease dynamics, to understand how it evolved, and predict what might come. Crucial results concern with the estimation of the so-called *basic reproduction number* (denoted by ℛ_0_), a metric which indicates the average number of secondary infections generated in a fully susceptible population by one infected host over the course of his infection. The basic reproduction number is a reference parameter in mathematical epidemiology used to understand if, and in which proportion, a disease will spread among the population.

Mathematical models were developed already for the largest epidemics reported before 2014. Chowell et al. [8] proposed a stochastic SEIR (susceptibles—exposed—infected—recovered) model to fit data from the 1995 and 2000 outbreaks in Congo and Uganda, respectively. They estimated that, in the absence of intervention, ℛ_0_ was 1.83 (with standard deviation 0.06) for Congo and 1.34 (with standard deviation 0.03) for Uganda. In [9], a quite similar stochastic SEIR model yields a lower estimate for Congo (ℛ_0_ ≈ 1.4). Data from the same epidemic were recently reconsidered by Ndanguza et al. [10].

The stochastic SEIR model in [8] was extended by Legrand et al. [11] with two more compartments, one for hospitalized and one for dead patients who are not yet buried. The basic reproduction number estimated with this model was 2.7 (95% Confidence Interval (CI): 1.9–2.8) for the 1995 epidemic in Congo, and 2.7 (95% CI: 2.5–4.1) for the 2000 epidemic in Uganda. Moreover, the authors studied various scenarios to estimate the effects of several parameters, such as the duration of the time to interventions, the hospitalization rate of Ebola cases after interventions, the efficacy of interventions at hospital. The basic reproduction number was split into three components, representing the virus transmission through non-hospitalized patients, hospitalized patients, and transmission following funeral attendance. Legrand’s compartmental model was used in [12] to revisit data from the first known Ebola outbreak (Congo, 1976), providing ℛ_0_ ≈ 1.34 (95% CI: 0.92–2.11).

The first mathematical models for 2014 EDV epidemics appeared around September of the same year. In [13], time series of weekly reported EVD cases in Guinea, Sierra Leone, and Liberia up to 8 September 2014, were used to estimate ℛ_0_ and its dynamical changes, affected by control measures. The data were fit with piecewise exponential curves. The basic reproduction number varied from 1.2 (95% CI: 1.0–1.3) in Sierra Leone, to 2.3 (95% CI: 1.8–2.8). Similar piecewise fit was provided in [14]. Althaus [15] uses a SEIR model with control measures beginning immediately after the appearance of the first infected case in a country, and estimates ℛ_0_ to be 1.51 for Guinea, 2.53 for Sierra Leone and 1.59 for Liberia.

Legrand’s model was applied to Ebola by Rivers et al. [16], who estimated ℛ_0_ to be approximately 2.2 for Sierra Leone and 1.78 for Liberia, fitting data from December 2013 to October 2014. The same model was used in [17] to consider spatial spread of Ebola and exportation of the virus to other countries. The results indicated that the outbreak was likely to spread further among African countries, increasing the risk of pandemic on a longer time scale.

Focus was made on the reported EVD cases and deaths in Montserrado County, Liberia [18–21]. Chowell et al. [20] used the logistic curve to fit the cumulative number of cases in Liberia, up to 23 October 2014. In [18], data from 7 July to 22 September 2014 were used to fit a stochastic model, which considers the level of infectiousness of ill patients or corpses. It was estimated that patients who did not survive the disease had the highest potential for transmitting the virus during disease progression.

In [21], a mathematical model was developed to investigate whether various intervention strategies, such as the distribution of protective kits to households, could have an effect on controlling the spread of Ebola virus in the country. The model parameters were obtained by fitting data of reported cases and deaths as of 8 October 2014, and ℛ_0_ was estimated to be 2.49 (95% CI: 2.38–2.60). The model predicted that with allocation of kits on 31 October 2014 there would have been between 46123 (95% CI: 37897–4295) and 78623 (95% CI: 71304–86442) EVD cases by 15 December 2014. Effects of further preventive measures were considered in [19].

Drake et al. [22] developed a multitype branching process model that incorporates heterogeneities and time-varying parameters to reflect changes in the human behavior as well as in the introduction of intervention strategies, e. g., in the rates of hospitalization, exposure of healthcare workers, and secure burial.

Following Legrand et al. [11], we propose a compartmental model for Ebola virus disease outbreak. Key components of our model are compartments for hospitalized patients and for patients who died of the disease but are not yet buried. Individuals in both compartments play an important role in the chain of Ebola virus transmission. We fit our model to weekly incidence data reported by WHO from 28 December 2013 to 3 October 2014 [23]. For this first phase of the epidemic, we estimate the basic reproduction number.

While knowing ℛ_0_ in the initial phase of the epidemic can help to understand the potential rate of the spread, more detailed analysis is needed to identify efficient control measures. Among the causes of 2014 EVD outbreaks are the delay in interventions and the reluctance in the community to undertake preventive measures. For this reason, in the second part of this work, we focus on the impact of intervention strategies which have been introduced to control the spread of the disease. Using time-dependent parameters to describe intervention strategies, we fit our model to weekly incidence data reported by WHO from 3 October 2014 to 13 February 2015 [7, 23, 24]. We derive a final size relation, valid from the time of intervention. This is an analytic formula that can be used to predict the total number of cases during the whole outbreak, providing a reliable approximation when interventions are effective. Performing sensitivity analysis, we study the effects of model parameters on the basic reproduction number and on the final size of the epidemic. In this way we identify factors playing a key role in the spread of Ebola virus, and intervention strategies resulting in effective control of the epidemic. To assess the impact of the timing of interventions, we simulate different scenarios with the same target control parameters, changing only the time of interventions.

As of February 2015, the number of new reported cases has significantly dropped, and it seems that the current level of intervention has effectively stopped the outbreak. The risk of infection might now be perceived to be lower, and this could induce community member and healthcare workers to relax protective measures. We use our model to consider changes in the community behavior and relaxation of interventions in the future, showing that ceasing current controls has the potential risk of a new Ebola outbreak.

## Methods

### Compartmental model

We develop a compartmental population model, following an earlier work by Legrand et al. [11]. Individuals are classified as follows. Susceptible individuals (S) are those who can be infected with Ebola virus. Exposed individuals (E) have been infected with Ebola virus, though they are not yet infectious, nor symptomatic. After a latent phase (about 10 days from infection [5]) the first symptoms appear and the exposed host becomes infective. We distinguish between infective Ebola patients in the community (I) and hospitalized patients (H). Patients who died of the disease and are not yet buried (D) are still carrying the virus and may transmit the disease during traditional funerals. Hosts who recovered from the infection are removed (R) from the chain of transmission.

**Fig 1:**
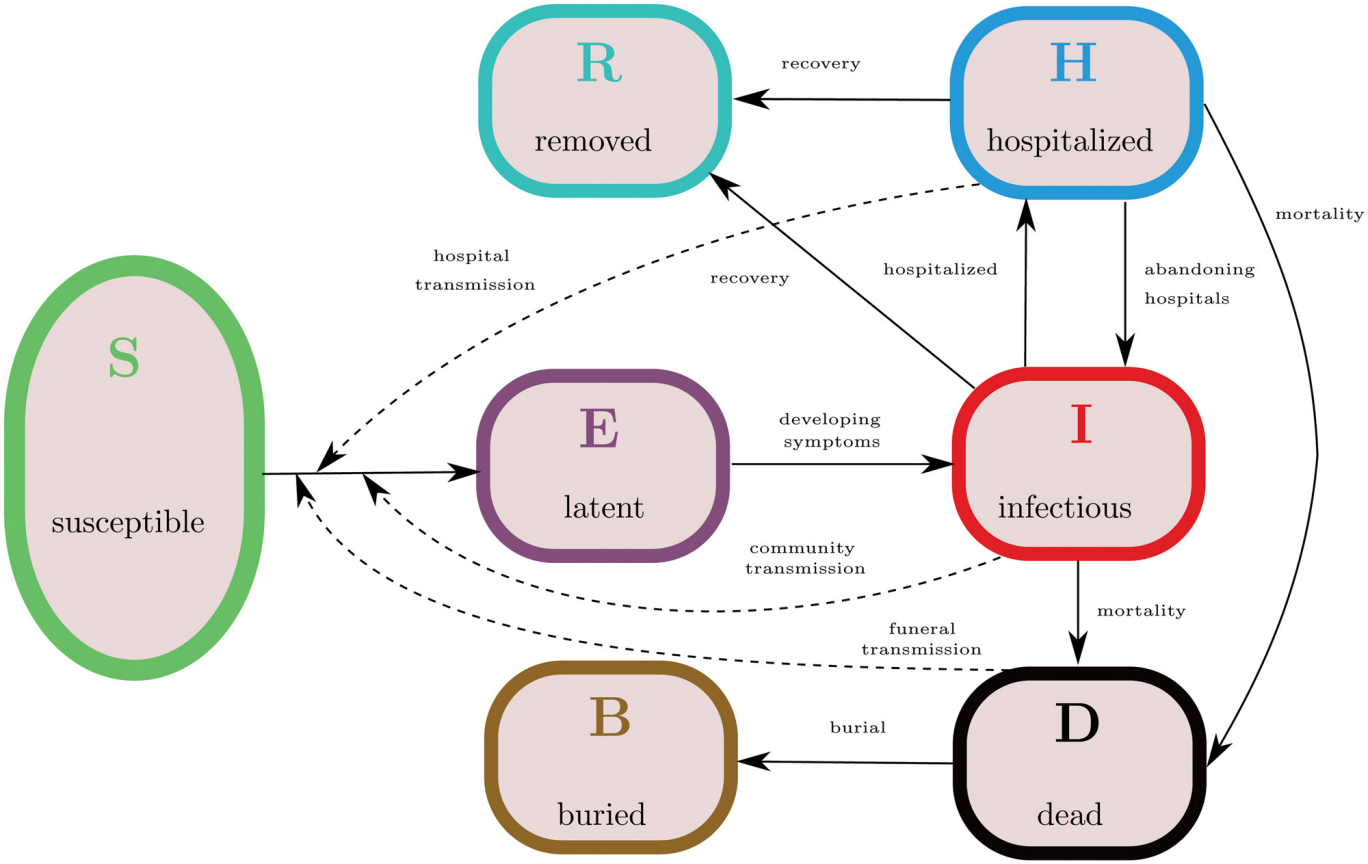
Model structure for 2014 Ebola outbreak in West Africa. Solid arrows indicate transition from one compartment to another, dashed arrows indicate virus transmission due to contact with infectives. The virus can be transmitted to susceptible hosts (S) from infectious patients in the community (I), hospitalized patients (H) or patients who died of the disease and are not yet buried (D). Upon infection, susceptible hosts enter a latent phase (E), in which they are not yet infective. After symptoms onset, they become infective and might be hospitalized, recover from the disease or die and remain infectious until buried (B). Hospitalized patients might abandon the healthcare facilities and return to community, otherwise they either recover or die of the disease. Hosts who recovered from the infection are removed (R) from the chain of transmission.

Transmission of Ebola virus is due not only to contact with infectives in the community, but also with hospitalized patients and dead Ebola patients. Indeed, a large part of infections occurred at hospitals and funerals with post-mortem contacts [6]. In this view, the compartments (H) and (D) are crucial components of our model. Our approach resembles the one in [11], with the extension that we include patients who abandon healthcare facilities and return to community, as it has been reported to happen in several cases [6]. We use two additional variables to track the cumulative number of cases (C) and burials (B). The transmission diagram of the model is depicted in Fig 1, and the governing equations are specified in Appendix A.1.

### Modeling the impact of intervention

To prevent and control the spread of Ebola, intervention strategies were adopted already during the 1995 Congo and the 2000 Uganda epidemics [8]. These include surveillance, isolation of suspected cases, information and instructions for the community, introduction of protective clothing for healthcare workers, and the rapid burial of patients who die from the disease [6]. Such control measures correspond to changes in certain model parameters. For example, an increasing hospital capacity shall correspond to increasing the hospitalization rate; education about Ebola transmission and distribution of protective kits for households might reduce the transmission rate in the community; definition of a more rigorous protocol for health facilities shall reduce transmission of the virus in the hospitals.

To describe the effect of intervention strategies in our model, we identify five control parameters, namely, the transmission rates in the community (*β*), in hospitals (*θ*) and at funerals (*φ*), the hospitalization rate (*η*) and the burial rate (*b*). Drake et al. [22] used the same control parameters to forecast the number of Ebola cases which should have been reported in the fall of 2014. We assume that, once intervention strategies have been introduced, control parameters are changing gradually in time. This is a realistic assumption, as intervention strategies are indeed hard to be applied when the community refuses to cooperate. WHO reports show that, as of February 2015, control indicators (such as the number of unsafe burial reported, the hospitalization rate and transmission rate in hospitals) are still far from the target [24].

We illustrate the structure of our time-dependent control parameters by mean of the transmission rate in the community. After intervention occurred at time *T*, this transmission rate gradually changes from value *β* to value $\tilde{\beta }$, with $\tilde{\beta }<\beta$, according to

$$\beta \left(t\right)=\{\begin{array}{cc} \beta & \text{for}\;t<T\\ \tilde{\beta }+\left(\beta -\tilde{\beta }\right)e^{-q_{\beta }\left(t-T\right)} & \text{for}\;t\geq T. \end{array}$$

The parameter *q*_*β*_ describes how fast the transition from *β* to $\tilde{\beta }$ occurs. The mean value $(\beta +\tilde{\beta })/2$ is reached at time *T*+*t*_*β*_, where *t*_*β*_ = ln(2)/*q*_*β*_. The same time-dependent transmission rate was previously introduced in [8]. Similarly, we assume that after intervention the contact rates *φ* and *θ* decrease from value *φ* to value $\tilde{\varphi }$ (respectively, from *θ* to $\tilde{\theta }$), whereas the hospitalization rate and the burial rate increase (from *η* to $\tilde{\eta }$, and from *b* to $\tilde{b}$, respectively).

### Parameter estimation and sensitivity

To isolate parameters of significant influence on the basic reproduction number, we perform parameter sensitivity analysis as follows. Latin Hypercube Sampling (LHS), an efficient statistical sampling method permitting simultaneous variation of the values of all input parameters [25], is used to generate a representative sample set of 10-tuples of parameters from the parameter ranges indicated in Table 1. Indeed, since the expression of ℛ_0_, see below, does not involve the incubation time 1/*α*, only the remaining ten parameter-ranges in Table 1 are considered in the Partial Rank Correlation Coefficients (PRCC) analysis for the basic reproduction number. Using PRCC we can rank the effect that each parameter has on the outcome, when other parameters are simultaneously varying in the given ranges. Calculation of PRCC allows to determine which statistical relationships exist between each input parameter and the outcome variable [26]. Parameters with PRCC larger than zero are positively correlated with 𝓡_0_, that is, the basic reproduction number increases as these parameter values are increased. Parameters with negative PRCC will decrease 𝓡_0_ as they are increased. Analogous parameter sensitivity analysis is performed on the final size relation, using the parameter ranges indicated in Table 2. With the help of PRCC we identify key intervention parameters and test their effects on the total number of hosts who have been infected during the course of the epidemic. The sensitivity of the final epidemic size to control parameters is also visualized in contour plots. Moreover, we use the time of intervention as a parameter and investigate the sensitivity of the epidemic outcome with respect to the delay in interventions.

**Table 1:** Parameter estimates for the 2014 Ebola outbreak. Parameter descriptions, fitted values and ranges used for parameter sensitivity analysis. Comparable values can be found in references indicated in the last column.

Parameter	Description	Fitted value (Range)	References
*β*	Transmission rate in the community before intervention (per week)	0.532 (0.350–0.575)	[11]
*θ*	Transmission rate in hospitals before intervention (per week)	0.328 (0.100–0.480)	[11]
*φ*	Transmission rate at funerals before intervention (per week)	2.104 (1.402–2.475)	[11]
1/*η*	Mean time from symptoms onset to hospitalization	4.8 (4.8–5.3) days	[5, 11, 17, 19]
1/*b*	Mean time from death to burial	5.4 (4–6.6) days	[6]
1/*γ*	Mean duration of the infection	10.4 (9.5–10.5) days	[6, 11, 17]
1/*α*	Mean duration of the incubation period	10 (9.5–10.5) days	[5, 19]
1/*γ*_*H*_	Average permanence in the hospital	4.6 (4.4–4.9) days	[19]
*δ*	Case fatality ratio in the community	73% (69%–73%)	[5, 11, 15]
*δ*_*H*_	Case fatality ratio in hospitals	61% (52%–64%)	[5]
*κ*	Hospital leaving rate (per week)	0.0025 (0.0022–0.0028)	

**Table 2:** Estimates for intervention parameters. Parameter descriptions, fitted values and ranges used for parameter sensitivity analysis. Comparable values can be found in references indicated in the last column.

Parameter	Description	Fitted value (Ranges)	Comments
$\tilde{\beta }$	Transmission rate in the community after intervention (per week)	0.505 (0.425–0.532)	Corresponds to 5% decrease in the transmission in the community after interventions, cf. [11]
*t*_*β*_	Time to achieve $(\beta +\tilde{\beta })/2$	12.5 days	
$\tilde{\theta }$	Transmission rate in hospitals after intervention (per week)	0.095 (0.033–0.328)	Corresponds to 71% decrease in the transmission in hospitals after interventions, cf. [11, 19]
*t*_*θ*_	Time to achieve $(\theta +\tilde{\theta })/2$	11.1 days	
$\tilde{\varphi }$	Transmission rate at funerals after intervention (per week)	1.115 (0.210–2.104)	Corresponds to 47% decrease in the transmission at funerals after interventions, cf. [11]
*t*_*φ*_	Time to achieve $(\varphi +\tilde{\varphi })/2$	10.3 days	
$1/\tilde{\eta }$	Mean time from symptoms onset to hospitalization	4.1 (2–5) days	cf. [11]
*t*_*η*_	Time to achieve $(\eta +\tilde{\eta })/2$	27.1 days	
$1/\tilde{b}$	Mean time from death to burial	4.9 (1–5.4) days	cf. [6]
*t*_*b*_	Time to achieve $(b+\tilde{b})/2$	21.2 days	

## Results

### Basic reproduction number and the early phase of the outbreak

To capture the characteristics of the early phase of the 2014 West Africa outbreak, first we identify the basic reproduction number (for the derivation, see Appendix A.2),

$${𝓡}_{0}=\underset{{𝓡}_{C}}{\underset{︸}{\frac{\beta (\gamma_{H}+\kappa )}{\gamma_{H}\eta +\gamma \left(\gamma_{H}+\kappa \right)}}}+\underset{{𝓡}_{H}}{\underset{︸}{\frac{\eta \theta }{\gamma_{H}\eta +\gamma \left(\gamma_{H}+\kappa \right)}}}+\underset{{𝓡}_{F}}{\underset{︸}{\frac{\varphi (\gamma_{H}\delta_{H}\eta +\gamma \delta \left(\gamma_{H}+\kappa \right))}{b(\gamma_{H}\eta +\gamma \left(\gamma_{H}+\kappa \right))}}},$$ (1)

with parameters as in Table 1. The basic reproduction number clearly breaks down to three components: secondary infections generated in the community (ℛ_*C*_), in hospitals (ℛ_*H*_), and at funerals (ℛ_*F*_). Similar components of ℛ_0_ were identified in previous works [11, 17, 18, 27].

**Fig 2:**
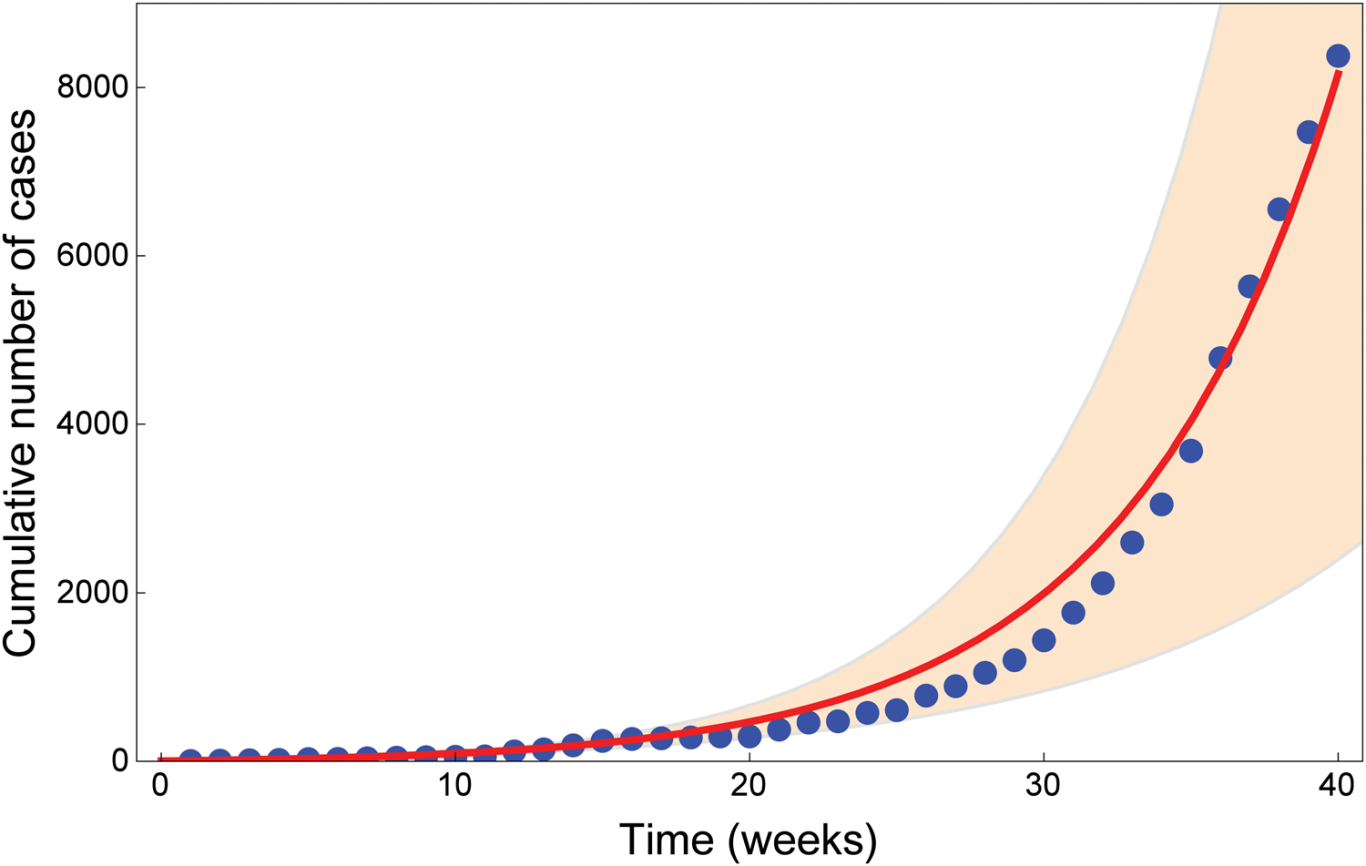
Data fit for Ebola cases in West Africa from week 1 to week 40. The blue dots show the cumulative number of cases reported by [23] from week 1 (ending 3 Jan 2014) to week 40 (ending 3 Oct 2014). The cumulative number of cases reported is the sum of confirmed, suspected and probable cases. The red curve shows the fit with parameter values in Table 1. The orange region shows the total number of cases predicted by the model (95% CI).

In order to obtain realistic parameter values, we fit our model solutions to the WHO dataset for weekly case incidence up to week 40 of 2014 (ending 3 October) [23]. The cumulative number of cases reported in Fig 2 is the sum of confirmed, suspected and probable cases. Same data were used, e.g., in [15, 22]. Starting from plausible ranges taken from the literature (see Table 1), we use LHS to generate 10000 sample parameter sets for our model. We run a numerical simulation for each sample and select the best fit, in the least-squares sense. Estimated parameter values up to week 40 are reported in Table 1. The best fit provides the value ℛ_0_ = 1.44 varying in the range 0.75–1.92, and matching estimated values in previous works [8, 12, 13]. Reported cumulative incidence data and numerical solution are depicted in Fig 2 together with the 95% confidence range, obtained by allowing for each parameter a 5% relative error with respect to the best fit.

Using the formula (1) and the estimated parameter values, we find that the three components of the basic reproduction number are ℛ_*C*_ = 0.25 (0.16–0.29), ℛ_*H*_ = 0.15 (0.04–0.23), and ℛ_*F*_ = 1.05 (0.48–1.49). This indicates that the most important factor for the spread of the epidemic is the virus transmission occurring during traditional burial practices. We conjecture that Ebola spread can be effectively controlled by a significant decrease in the funeral reproduction number, while control by other measures is not likely to stop the outbreak, as long as funeral linked transmissions remain high. For the 1995 Ebola epidemic in Congo, Legrand et al. [11] estimated ℛ_*C*_ = 0.5, ℛ_*H*_ = 0.4, ℛ_*F*_ = 1.8, confirming funerals as the key factor for virus spread. In contrast, Gomes et al.[17] estimated ℛ_*C*_ = 0.8, ℛ_*H*_ = 0.4, and ℛ_*F*_ = 0.6, implying that the virus mostly transmitted in the community. Remarkably, in our work as in previous ones, the lowest component of ℛ_0_ is the one related to secondary infections generated in hospitals.

**Fig 3:**
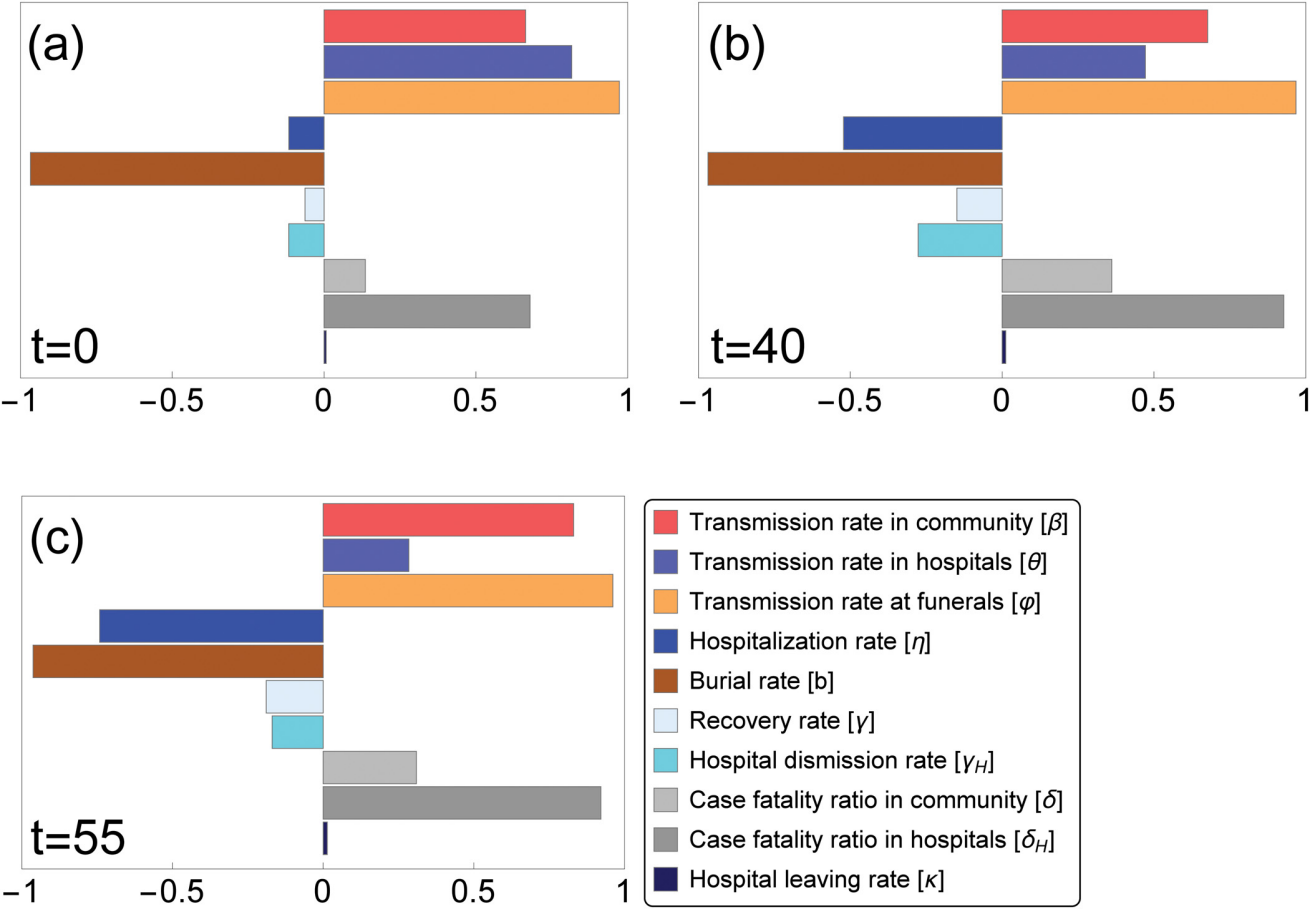
Partial rank correlation coefficients (PRCCs) of the ten parameters that influence disease spread. Parameter values from the ranges in Tables 1 and 2 are considered. Parameters with PRCC larger than zero are positively correlated with 𝓡_eff_(*t*), that is, the effective reproduction number at time *t* increases as these parameter values are increased. Parameters with negative PRCC will decrease 𝓡_eff_(*t*) as they are increased. Variations in the burial rate and in the transmission rate at funerals have the greatest effect on the reproduction number, in particular at the beginning (*t* = 0) of the epidemic (a). Fig (b) and (c) show how the relative importance of the parameters on the effective reproduction number changes in time.

In order to study sensitivity of the basic reproduction number to parameter variations, we perform PRCC analysis on our sample set (parameter ranges in Table 1). The result, visualized in Fig 3(a), confirms that the most important parameters in reducing ℛ_0_ are, indeed, the funeral transmission rate and the time to burial. On the other hand, the hospital abandonment rate has not much influence on ℛ_0_ in these parameter ranges.

### Assessment of the interventions

**Fig 4:**
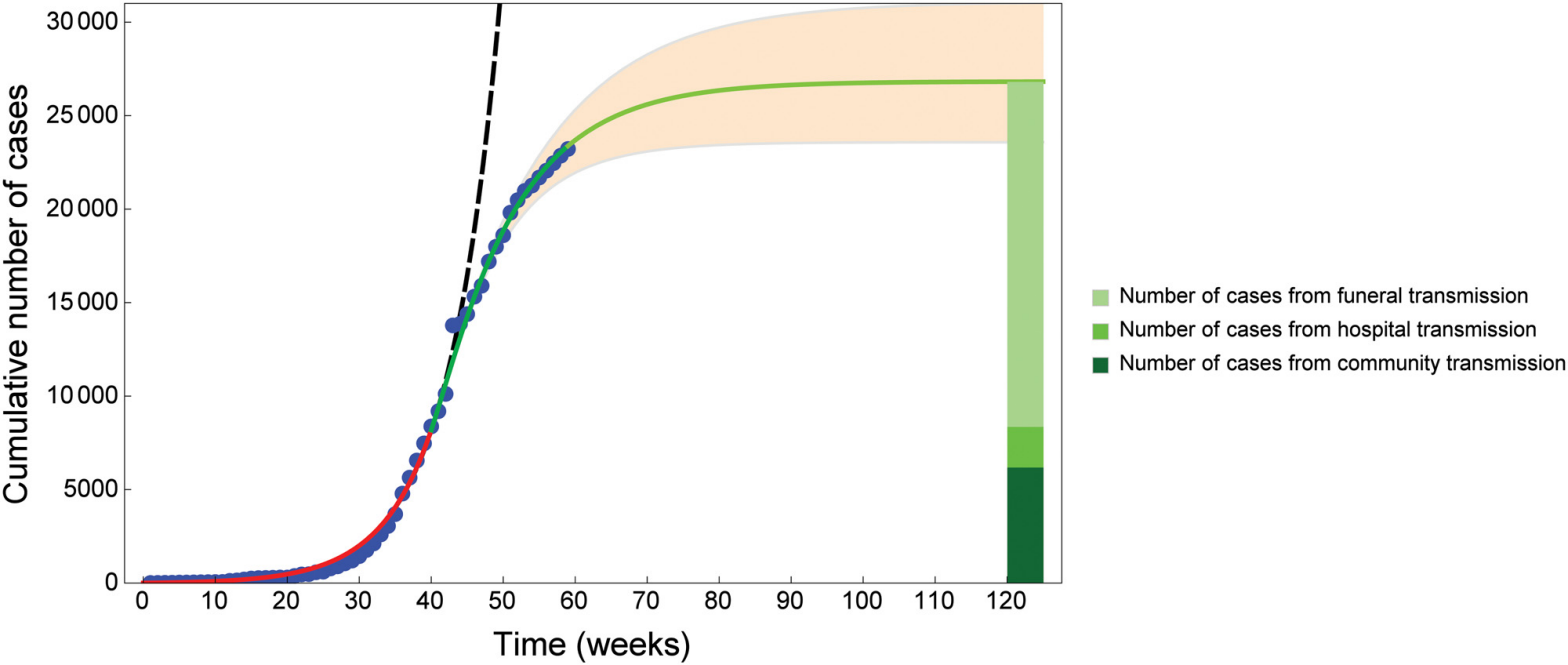
Data fit for Ebola cases in West Africa from week 1 to week 60 and prediction until week 120. The blue dots show the cumulative number of cases reported by [7, 23, 24]. The cumulative number of cases reported in Fig 2 is the sum of confirmed, suspected and probable cases. The red curve shows the fit obtained with parameter values in Table 1. At week 40 (ending 3 Oct 2014) intervention strategies are introduced. The green curve shows the model fit from week 40 to week 59 (ending 13 Feb 2015), with parameter values in Table 2. The light green curve shows model prediction until week 120 (ending 15 April 2016). The orange region shows the model-predicted total number of cases (95% CI) from the introduction of intervention strategies. The green charts on the right shows which proportion of the estimated cases at week 120 are due to contacts in the community (23%, 6191 out of 26809 cases), contacts in the hospitals (8%, 2173 out of 26809 cases) and contacts at funerals (69%, 18445 out of 26809 cases). If intervention strategies are not introduced at week 40 (black dashed curve), the numerical solution curve deviates from the real data considerably.

Model predictions with parameter values in Table 1 yield a reasonably good fit in the *early phase* of the epidemic, up to week 40. However, after week 40, the numerical solution curve deviates from the real data considerably (see the black dashed curve in Fig 4). This is due to the fact that parameter values in Table 1 do not take into account control measures achieved thanks to national and international public health efforts. In order to capture the dynamics of the *intervention phase* of the epidemic, we extended the initial model to account for control measures. As described in the Methods Section, we select five target control parameters: the transmission rate in the community (*β*), the transmission rate in hospital (*φ*), the transmission rate at funerals (*θ*), the hospitalization rate (*η*) and the time to burial (*b*). After intervention strategies have been introduced, these intervention parameters change gradually in time from a baseline parameter value to a target value. Baseline parameters are chosen according to the best fit for the early phase (Fig 2). Target parameter values are estimated fitting our model solutions to weekly case incidence reported by [7, 23, 24] from week 40 to week 59 (ending 13 Feb 2015).

As for the early phase of the epidemic, we use LHS to generate sample parameter sets for our model (ranges in Table 2), run a numerical simulation for each sample and select the best fit. Estimated parameter values for the intervention phase are reported in Table 2, indicating a 5% reduction in the transmission rate in the community, a 71% reduction in the transmission rate in hospitals, and a 47% reduction in the transmission rate at funerals. On the other side, the mean time to hospitalization decreased from 4.8 to 4.1 days (corresponding to higher hospitalization rate) and the mean time from death to burial decreased from 5.4 to 4.9 days (corresponding to increased burial rate). These estimates mostly agree with the results in [11, 19], whereas Legrand et al [11] estimated a decrease of 75–100% for the 1995 and 2000 epidemics in Congo and Uganda, respectively.

Fig 4 shows the best fit from week 40 to week 59, as well as predictions until week 120 (ending 15 April 2016), and the 95% confidence range, obtained by allowing for each parameter a 5% relative error with respect to the best fit. Given current intervention measures, the cumulative number of cases is predicted to be around 26800 until week 120 (95% CI: 23580–31030). Out of these, we estimate ca. 23% of the cases to be due to contacts in the community, ca. 8% to follow contacts in the hospitals, and ca. 69% to come from burial practices. WHO estimated that in Guinea and Sierra Leone, 60–80% of the cases are linked to traditional funerals [6].

As time elapses, the dynamics of the outbreak together with the introduction of control measures shift the relative importance of the parameters on the disease spread, as it is shown in Fig 3b and 3c.

### Final epidemic size after intervention

By mathematical analysis, we derived a final size relation after interventions. This is an analytic formula that can be used to predict the total number of cases during the whole outbreak, providing a reliable approximation when the total population and the parameters do not change much after some time *T*, as this is the case when the interventions are effective. The final size relation is computed in Appendix A.3 as

$$\begin{array}{cc} \ln S_{T}-\ln S_{\infty }= & \frac{{𝓡}_{c}}{S_{T}}(N_{T}-S_{\infty })-\frac{{𝓡}_{c}}{S_{T}}R_{T}\\ & +\frac{\tilde{\varphi }}{\tilde{b}N_{T}}D_{T}+H_{T}(\frac{{𝓡}_{c}}{S_{T}}(1-\frac{z}{y}+\frac{xv}{yu})-\frac{v}{uN_{T}}). \end{array}$$ (2)

**Fig 5:**
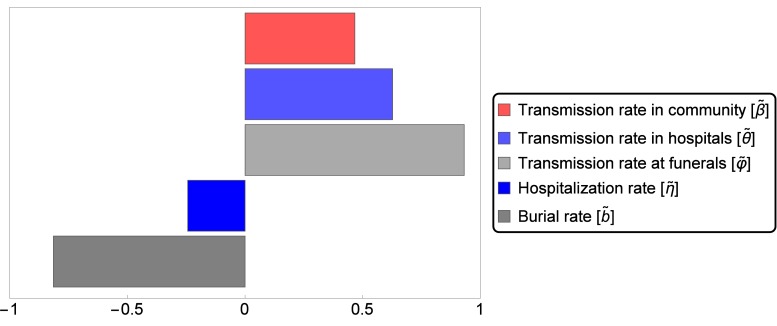
Partial rank correlation coefficients (PRCCs) of the five intervention parameters that influence the cumulative number of cases. Parameter values from the ranges in Table 2 are considered. Parameters with PRCC larger than zero are positively correlated with the total number of hosts who have been infected over the course of the epidemic. Parameters with negative PRCC will decrease the number of hosts who have been infected over the course of the epidemic, as they are increased. The greatest effect will be observed with variations in the burial rate, in the transmission rate at funerals and in the transmission rate in hospitals.

**Fig 6:**
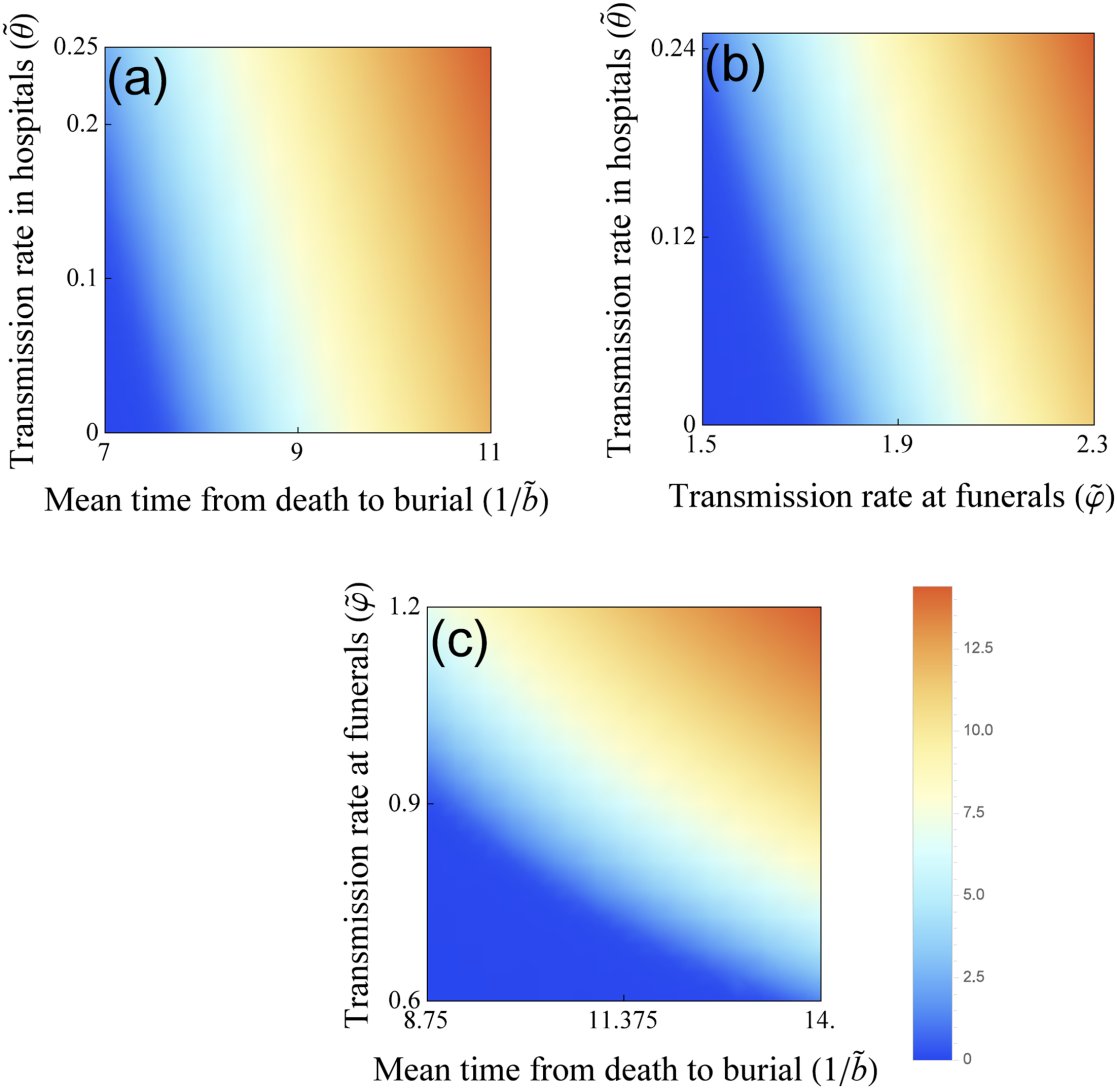
The effects of intervention on the cumulative number of cases. Sensitivity analysis of the total number of cases during the course of the epidemic. The key intervention parameters, namely, the transmission rate at funerals (*φ*), the transmission rate in hospitals (*θ*) and the time from death to burial (1/*b*) help to control the attack rate of the epidemic. Blue area corresponds to lower cumulative number of cases, the orange area corresponds to higher cumulative number of cases.

Here the subindex *T* refers to the size of the given compartment at intervention time *T*, in particular *N*_*T*_ is the population size at time *T*. Further, ℛ_*c*_ is the effective reproduction number at time *T*,

$${𝓡}_{c}=(\frac{\tilde{\varphi }u}{\tilde{b}}+y)\frac{1}{u+x}\cdot \frac{S_{T}}{N_{T}},$$

and

$$\begin{array}{cc} x & =\tilde{\eta }\left(1-\delta_{H}\right)\gamma_{H}+\left(\gamma_{H}+\kappa \right)\left(1-\delta \right)\gamma ,\;z=\left(1-\delta_{H}\right)\gamma_{H}\tilde{\beta }-\left(1-\delta \right)\gamma \tilde{\theta },\\ y & =\tilde{\eta }\tilde{\theta }+\left(\gamma_{H}+\kappa \right)\tilde{\beta },\;u=\tilde{\eta }\delta_{H}\gamma_{H}+\left(\gamma_{H}+\kappa \right)\delta \gamma ,\;v=\delta_{H}\gamma_{H}\tilde{\beta }-\delta \gamma \tilde{\theta }, \end{array}$$

with the parameters in Table 2. The notation *S*_∞_ expresses the number of susceptibles at the end of the outbreak. Thus, the total number of cases can be obtained as *S*_0_−*S*_∞_, where *S*_0_ is the number of susceptibles at time 0. Having an analytic formula for the final size, we can examine the sensitivity of the total number of cases to the intervention parameters. The relative importance is shown by means of PRCC in Fig 5, while the sensitive region is plotted on two-parameter planes in Fig 6. We conclude that the most important parameters in reducing the final size are the funeral transmission rate and the time to burial.

### Impact of the timing of interventions

**Fig 7:**
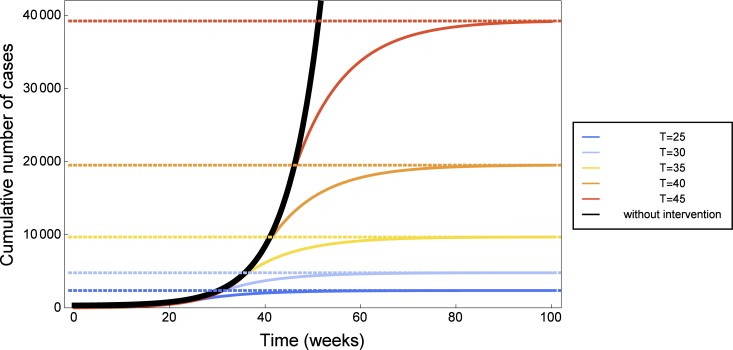
The importance of timely intervention in 2014 Ebola outbreak. Predicted number of cumulative cases: numerical simulations of the mathematical model (solid lines) and values estimated by the final size formula (2) (dashed lines). The later intervention occurs, the higher the number of Ebola cases. Simulations were done for hypothetical and immediately effective intervention at week 25 (blue), week 30 (light blue), week 35 (yellow), week 40 (orange), week 45 (red). If no intervention strategies are introduced (black curve), the number of Ebola cases grows exponentially.

To assess the impact of the timing of interventions, we investigate sensitivity of the solutions with respect to the time of intervention *T*. We assume intervention strategies are optimally introduced at time *T*. This means that there is no smooth transition from the baseline parameter to the target parameter, but rather an instantaneous switch at time *T*, which approximates very large values of exponents *q*_*β*_, *q*_*φ*_, *q*_*θ*_, *q*_*η*_, *q*_*b*_. Hence we use the baseline parameters from Table 1 up to time *T*, then fix the intervention parameters as in Table 2 and use them in the model equations after time *T*. Then we let *T* vary. Fig 7 shows simulations for intervention at weeks 25, 30, 35, 40, 45, as well as the case of no intervention, alongside with the predicted value of the total cases using our final size relation. One can see that for such parameter ranges, the final size relation is indeed very accurate. The black curve in Fig 7 corresponds to model prediction with baseline parameters and indicates that, in the absence of control measures, Ebola reported cases would have grown exponentially in this time frame. The orange curve in the same figure, corresponding to intervention at week 40, can be compared to reported cases and to the fit in Fig 4. We observe that if control strategies gradually applied from week 40 were immediately effective, there would have been less than 22000 reported cases, instead of the 26800 indicated in Fig 4. All in all, we find that the final epidemic size is very sensitive to the timing of interventions: five weeks delay can roughly double the total number of cases.

## Discussion

The 2014 epidemic in West Africa has been the most severe Ebola virus disease outbreak ever reported. Several mathematical models have been developed to predict how the disease evolves, and to assess strategies for mitigation and control. Our contribution aimed to understand the dynamics of the 2014 EVD outbreak, taking into consideration the continuously changing control measures adopted by African institutions and international organizations. We proposed a deterministic model based on the work by Legrand et al. [11], which is an extension of the SEIR model. Classifying infective agents into three groups, the model kept track of infections occurred in the community, in hospitals or during traditional funerals. To capture the key characteristics of the initial growth phase, we fitted our model to weekly incidence data reported by [23] for the first 40 weeks of 2014, until 3 October. Parameter values were estimated by generating sample sets from previously proposed parameter ranges. For the first phase of the epidemic, we estimated ℛ_0_ to be 1.44, in accordance with previously estimated values [8, 12, 13]. The basic reproduction number could be split into three components, for the virus transmission through non-hospitalized patients, hospitalized patients, and transmission following funeral attendance. Similarly to the results in [11, 18], we found that the most important factor for the spread of the epidemic was virus transmission during traditional burial practices. PRCC analysis confirmed that funeral transmission rate and time to burial are the most important parameters in reducing ℛ_0_ (Fig 3).

In accordance with WHO reports [6], we identified 10 October 2014 as the time at which control strategies were introduced. For the *intervention phase* we selected five control parameters (*β*, *φ*, *θ*, *η* and *b*) which change gradually in time. The model was fitted to weekly incidence to data from October 2014 to February 2015. The best fit suggested that control measures could effectively reduce the transmission rate in hospitals (*θ*) and the transmission rate at funerals (*φ*), whereas the transmission rate in the community (*β*) was barely affected. Indeed, the observation of a stricter protocol in healhcare facilities as well as safe burial practices are most likely to be controlled than community behaviors and interactions. The fitted control parameter values could be compared with WHO indicators [24] for intervention measures, such as the time from symptom onset to isolation, the number of reported safe burials, or the number of districts in which the community was reluctant to cooperate. The WHO report [24] stated that months after interventions, control indicators were shown to be yet far from the target. In accordance, we found that certain control parameters, such as *η* or *b*, needed 40 to 60 days to switch from the baseline value to the intervention target value. Using the fitted intervention parameters, the model could predict about 26800 total cumulative cases until Arpil 2016 (Fig 4), out of which ca. 70% would come from burial practices. This result matches WHO estimates [6].

It is not an easy task to predict the final size of an ongoing epidemic [20]. From the time of intervention on, our final size relation (2) holds. Its computation is an innovative result for Ebola disease models, which has not been presented before and will be useful for the investigations of possible outbreaks in the future as well. The analytic formula (2) is a reliable instrument for predictions on the total number of cases during the whole outbreak. The final size relation was also used to show that the burial rate, the transmission rate at funerals and the transmission rate in hospitals are key factors in controlling the total number of cases.

In the last part of the work we investigated the importance of timely intervention, showing that few weeks delay can result into twice as large total number of cases.

Using a relatively simple deterministic compartmental model naturally has several limitations. We assumed homogeneous mixing for the whole West Africa region, while in reality spatially heterogeneous epidemic patterns arose. Especially in the initial phase of the epidemics, there was much uncertainty regarding the reliability of data collection.

The quality of reporting and surveillance might have changed significantly as the epidemic progressed, but the real number of Ebola cases is still difficult to estimate. Here we have considered the reported number of confirmed, probable, and suspected Ebola cases (for the definitions, see [28]) from official WHO reports, as it was done in [15, 22]. Though we have not accounted for underreporting and other unreliabilities, our confidence bands cover a plausible range that likely captures the real trends.

**Fig 8:**
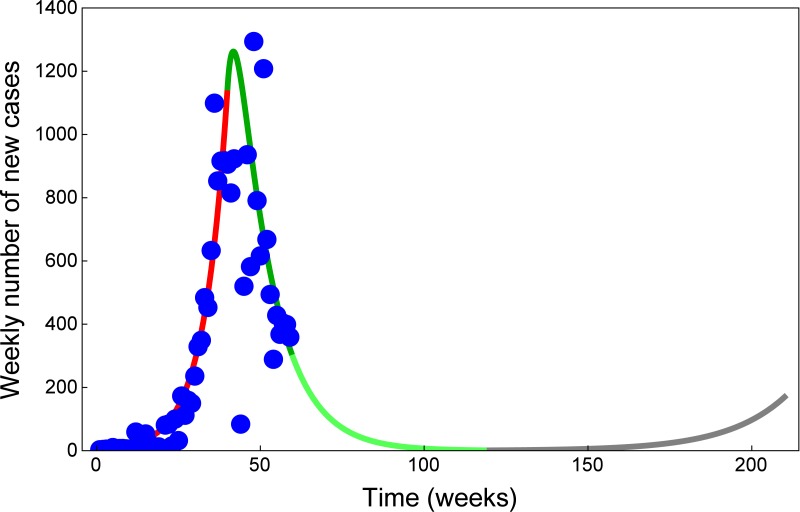
A new Ebola outbreak is possible if control is relaxed. Model fit for the number of new weekly reported cases, corresponding to the cumulative cases in Fig 4. The blue dots show new weekly cases reported by [7, 23, 24]. The red curve shows the fit obtained with parameter values in Table 1 up to week 40 (ending 3 Oct 2014). The dark green curve shows the model fit from the introduction of control measures at week 40 to week 59 (ending 13 Feb 2015), with parameter values in Table 2. The light green curve shows model prediction until week 200 (ending 27 October 2017). The gray curve shows the model prediction assuming that, at week 120 (ending 15 April 2016), intervention strategies are relaxed to the following values: $\tilde{\beta }=0.521$, $\tilde{\theta }=0.235$, $\tilde{\phi }=1.708$, $1/\tilde{b}=5.2$ and $1/\tilde{\eta }=4.6$.

Timely intervention strategies played a key role for the dynamics of the 2014 Ebola outbreak in West Africa. As of February 2015, the number of new reported cases has significantly dropped, and it seems that the current level of intervention has effectively stopped the outbreak. As the Ebola epidemic appears to be over, the risk of infection might now be perceived to be lower. This could induce community members and healthcare facilities to relax protective measures, for example, by reintroducing traditional burial practices. Relaxation of control measures can be easily incorporated into our compartmental model by time variance of the relevant parameters. Such a scenario is shown in Fig 8, using our current fits to weekly cases together with a future scenario in the case of relaxed control measures. This result shows that dismissing current intervention measures has the potential risk of a second outbreak. The phenomenon of such a resurgence has already been observed on local levels: in late November, several areas in Guinea that had been reporting no new cases were affected again [6]. We stress that protective measures among the community and healthcare workers shall not be relaxed too early. Since the majority of the population in the affected regions are still susceptible, as long as the virus was not eradicated, the risk of a new outbreak would persist.

## A Appendix

### A.1 The governing equations

In accordance with the structure diagram in Fig 1 and the description of the parameters in Table 1, the governing equations of the compartmental system can be written as

$$\begin{array}{cc} S^{\prime }\left(t\right) & =\;-\lambda (t)S(t),\\ E^{\prime }\left(t\right) & =\;\lambda (t)S(t)-\alpha E(t),\\ I^{\prime }\left(t\right) & =\;\alpha E(t)-\gamma I(t)-\eta I(t)+\kappa H(t),\\ R^{\prime }\left(t\right) & =\;\left(1-\delta \right)\gamma I\left(t\right)+\left(1-\delta_{H}\right)\gamma_{H}H\left(t\right),\\ D^{\prime }\left(t\right) & =\;\delta \gamma I\left(t\right)+\delta_{H}\gamma_{H}H\left(t\right)-bD\left(t\right),\\ H^{\prime }\left(t\right) & =\;\eta I\left(t\right)-\gamma_{H}H\left(t\right)-\kappa H\left(t\right), \end{array}$$ (3)

where the force of infection is

$$\lambda \left(t\right)=\beta \frac{1}{N(t)}I\left(t\right)+\varphi \frac{1}{N(t)}D\left(t\right)+\theta \frac{1}{N(t)}H\left(t\right),$$

and *N*(*t*) = *S*(*t*)+*E*(*t*)+*I*(*t*)+*R*(*t*) denotes the total population in the community. Initial value for the system (3) shall be nonnegative values.

This system is analogous to earlier works [11, 12, 16], with the addition of *κ* representing the rate at which patients abandon hospitals. To facilitate the analysis, we consider the auxiliary equations

$$\begin{array}{l} B^{\prime }\left(t\right)=bD\left(t\right)\\ C^{\prime }\left(t\right)=\alpha E\left(t\right), \end{array}$$

to monitor the cumulative number of burials and the cumulative number of cases.

### A.2 Derivation of the basic reproduction number

To calculate the basic reproduction number, we apply the method established by Diekmann et al. [29]. In this way we obtain ℛ_0_ number as the dominant eigenvalue of the next generation matrix *FV* ^−1^, where *F* is the transmission matrix and −*V* is the transition matrix of the infection subsystem at the disease-free state of the population. In our case, these matrices are

$$F=(\begin{array}{cccc} 0 & \beta & \varphi & \theta \\ 0 & 0 & 0 & 0\\ 0 & 0 & 0 & 0\\ 0 & 0 & 0 & 0 \end{array})\;\text{and}\;V=(\begin{array}{cccc} \alpha & 0 & 0 & 0\\ -\alpha & \gamma +\eta & 0 & -\kappa \\ 0 & -\delta \gamma & b & \delta_{H}\gamma_{H}\\ 0 & -\eta & 0 & \kappa +\gamma_{H} \end{array}).$$

Hence, the next generation matrix takes the form

$$(\begin{array}{cccc} {𝓡}_{0} & * & * & *\\ 0 & 0 & 0 & 0\\ 0 & 0 & 0 & 0\\ 0 & 0 & 0 & 0 \end{array}),$$

where

$${𝓡}_{0}=\frac{\eta \theta }{\gamma_{H}\eta +\gamma \left(\gamma_{H}+\kappa \right)}+\frac{\beta (\gamma_{H}+\kappa )}{\gamma_{H}\eta +\gamma \left(\gamma_{H}+\kappa \right)}+\frac{\varphi (\gamma_{H}\delta_{H}\eta +\gamma \delta \left(\gamma_{H}+\kappa \right))}{b(\gamma_{H}\eta +\gamma \left(\gamma_{H}+\kappa \right))}$$

and asterisks denote the entries of the next generation matrix which do not influence the dominant eigenvalue. Since our parameters can be time-dependent, the effective reproduction number can be expressed as

$${𝓡}_{\text{eff}}\left(t\right)={𝓡}_{0}\left(t\right)\frac{S(t)}{N(t)},$$

where ℛ_0_(*t*) means that we substitute the parameter values at time *t* into the ℛ_0_ formula above.

### A.3 Epidemic final size with intervention

In this section we assume that effective interventions are in place from time *T*, and for *t* > *T* our control parameters have the fixed values $\tilde{\beta },\tilde{\varphi },\tilde{\theta },\tilde{\eta },\tilde{b}$. To obtain the final size relation (2), first we show the existence of two invariants (first integrals) for system (3) under the assumption that *N* is constant for *t* ≥ *T*, denoted by *N*_*T*_: = *N*(*T*). First, we point out that all model variables remain nonnegative. Indeed, e.g., if for some time $\bar{t}>0$, the number of infectives $I(\bar{t})$ is zero while all other variables are nonnegative, then $I^{\prime }(\bar{t})=\alpha E(t)+\kappa H(t)\geq 0$. Similar considerations on the other variables show that, given nonnegative initial data, the solution of the system remain nonnegative.

As next we show that the populations in the compartments *E*, *I*, *D* and *H* all die out as *t* → ∞. To see that *E*(*t*) → 0 as *t* → ∞, we consider

$${\left(S\left(t\right)+E\left(t\right)\right)}^{\prime }=-\alpha E\left(t\right).$$

Obviously, if *E*(*t*) remains positive, then *S*(*t*)+*E*(*t*) drops below 0, which contradicts what shown above. To see that the compartments *I* and *H* die out, consider

$${\left(I\left(t\right)+H\left(t\right)\right)}^{\prime }=\alpha E\left(t\right)-\gamma I\left(t\right)-\gamma_{H}H\left(t\right).$$

Again, it is easy to see that if either *I*(*t*) or *H*(*t*) does not tend to 0 then *I*(*t*)+*H*(*t*) drops below 0 which is not possible. The statement for compartment *D* follows from the previous assertions. Let us define the quantities

$$\begin{array}{ll} x & ≔\;\tilde{\eta }\left(1-\delta_{H}\right)\gamma_{H}+\left(\gamma_{H}+\kappa \right)\left(1-\delta \right)\gamma ,\\ y & ≔\;\tilde{\eta }\tilde{\theta }+\left(\gamma_{H}+\kappa \right)\tilde{\beta },\\ z & ≔\;\left(1-\delta_{H}\right)\gamma_{H}\tilde{\beta }-\left(1-\delta \right)\gamma \tilde{\theta },\\ u & ≔\;\tilde{\eta }\delta_{H}\gamma_{H}+\left(\gamma_{H}+\kappa \right)\delta \gamma ,\\ v & ≔\;\delta_{H}\gamma_{H}\tilde{\beta }-\delta \gamma \tilde{\theta }, \end{array}$$

and the functions

$$\begin{array}{l} V_{1}\left(t\right)=\;x\;\text{ln}\;S\left(t\right)+\frac{y}{N_{T}}R\left(t\right)+\frac{z}{N_{T}}H\left(t\right)+\frac{\tilde{\varphi }}{\tilde{b}N_{T}}xB\left(t\right),\\ V_{2}\left(t\right)=\;u\;\text{ln}\;S\left(t\right)+\frac{y}{N_{T}}D\left(t\right)+\frac{v}{N_{T}}H\left(t\right)+\left(\frac{\tilde{\varphi }}{\tilde{b}N_{T}}u+\frac{y}{N_{T}}\right)B\left(t\right). \end{array}$$

To show that *V*_1_(*t*) and *V*_2_(*t*) are invariants for *t* ≥ *T*, we calculate the derivatives along the solutions of (3) to find, indeed,

$$\begin{array}{ll} V_{1}^{\prime }\left(t\right) & =\left[\tilde{\eta }\left(1-\delta_{H}\right)\gamma_{H}+\left(\gamma_{H}+\kappa \right)\left(1-\delta \right)\gamma \right](-\frac{\tilde{\beta }}{N_{T}}I\left(t\right)-\frac{\tilde{\varphi }}{N_{T}}D\left(t\right)-\frac{\tilde{\theta }}{N_{T}}H\left(t\right))\\ & \;+[\tilde{\eta }\frac{\tilde{\theta }}{N_{T}}+\left(\gamma_{H}+\kappa \right)\frac{\tilde{\beta }}{N_{T}}]\left(\left(1-\delta \right)\gamma I\left(t\right)+\left(1-\delta_{H}\right)\gamma_{H}H\left(t\right)\right)\\ & \;+[\left(1-\delta_{H}\right)\gamma_{H}\frac{\tilde{\beta }}{N_{T}}-\left(1-\delta \right)\gamma \frac{\tilde{\theta }}{N_{T}}]\left(\tilde{\eta }I\left(t\right)-\gamma_{H}H\left(t\right)-\kappa H\left(t\right)\right)\\ & \;+\frac{\tilde{\varphi }}{\tilde{b}N_{T}}\left[\tilde{\eta }\left(1-\delta_{H}\right)\gamma_{H}+\left(\gamma_{H}+\kappa \right)\left(1-\delta \right)\gamma \right]\tilde{b}D\left(t\right)\\ & =0 \end{array}$$

and

$$\begin{array}{ll} V_{2}^{\prime }\left(t\right) & =\;\left[\tilde{\eta }\delta_{H}\gamma_{H}+\left(\gamma_{H}+\kappa \right)\delta \gamma \right]\left(-\frac{\tilde{\beta }}{N_{T}}I\left(t\right)-\frac{\tilde{\varphi }}{N_{T}}D\left(t\right)-\frac{\tilde{\theta }}{N_{T}}H\left(t\right)\right)\\ & \;+\left[\tilde{\eta }\frac{\tilde{\theta }}{N_{T}}+\left(\gamma_{H}+\kappa \right)\frac{\tilde{\beta }}{N_{T}}\right]\left(\delta \gamma I\left(t\right)+\delta_{H}\gamma_{H}H\left(t\right)-\tilde{b}D\left(t\right)\right)\\ & \;+\left[\delta_{H}\gamma_{H}\frac{\tilde{\beta }}{N_{T}}-\delta \gamma \frac{\tilde{\theta }}{N_{T}}\right]\left(\tilde{\eta }I\left(t\right)-\gamma_{H}H\left(t\right)-\kappa H\left(t\right)\right)\\ & \;+\left(\frac{\tilde{\varphi }}{\tilde{b}N_{T}}\left[\tilde{\eta }\delta_{H}\gamma_{H}+\left(\gamma_{H}+\kappa \right)\delta \gamma \right]+\tilde{\eta }\frac{\tilde{\theta }}{N_{T}}+\left(\gamma_{H}+\kappa \right)\frac{\tilde{\beta }}{N_{T}}\right)\tilde{b}D\left(t\right)\\ & =\;0. \end{array}$$

In the sequel, for the sake of simplicity, we write *S*_*T*_ for *S*(*T*) and similarly for each compartment. In this notation, the control reproduction number ℛ*_c_*: = ℛ_eff_(*T*) is

$${𝓡}_{c}=(\frac{\tilde{\varphi }u}{\tilde{b}}+y)\frac{1}{u+x}\cdot \frac{S_{T}}{N_{T}}.$$ (4)

We calculate the final epidemic size from the time of interventions. From the invariance of *V*_1_(*t*) and *V*_2_(*t*), we have that *V*_1_(*T*) = *V*_1_(∞) and *V*_2_(*T*) = *V*_2_(∞). Further, *E*_∞_ = 0, *I*_∞_ = 0, *D*_∞_ = 0, and *H*_∞_ = 0, so we have

$$x\ln S_{T}+\frac{y}{N_{T}}R_{T}+\frac{z}{N_{T}}H_{T}+\frac{\tilde{\varphi }}{\tilde{b}N_{T}}xB_{T}=x\ln S_{\infty }+\frac{y}{N_{T}}R_{\infty }+\frac{\tilde{\varphi }}{\tilde{b}N_{T}}xB_{\infty },$$ (5)

$$u\ln S_{T}+\frac{y}{N_{T}}D_{T}+\frac{v}{N_{T}}H_{T}+(\frac{\tilde{\varphi }}{\tilde{b}N_{T}}u+\frac{y}{N_{T}})B_{T}=u\ln S_{\infty }+(\frac{\tilde{\varphi }}{\tilde{b}N_{T}}u+\frac{y}{N_{T}})B_{\infty }.$$ (6)

Multiply Eq (5) by *u*/*x*,

$$u\ln S_{T}+\frac{yu}{xN_{T}}R_{T}+\frac{zu}{xN_{T}}H_{T}+\frac{u\tilde{\varphi }}{\tilde{b}N_{T}}B_{T}=u\ln S_{\infty }+\frac{yu}{xN_{T}}R_{\infty }+\frac{u\tilde{\varphi }}{\tilde{b}N_{T}}B_{\infty }$$ (7)

and subtract Eq (6) from Eq (7):

$$\frac{uy}{xN_{T}}R_{T}+(\frac{zu}{xN_{T}}-\frac{v}{N_{T}})H_{T}-\frac{y}{N_{T}}D_{T}-\frac{y}{N_{T}}B_{T}=\frac{yu}{xN_{T}}R_{\infty }-\frac{y}{N_{T}}B_{\infty }$$ (8)

For simplicity of notation, let us define

$$Q≔Q_{T}=\frac{yu}{x}R_{T}+(\frac{zu}{x}-v)H_{T}-yD_{T}-yB_{T},$$ (9)

and the total number of individuals at time *T*

$$M≔M_{T}=N_{T}+D_{T}+B_{T}+H_{T}.$$ (10)

Then, relation Eq (8) implies

$$B_{\infty }=\frac{u}{x}R_{\infty }-\frac{Q}{y}.$$ (11)

Further, from the invariance properties we have that *M* = *R*_∞_ + *S*_∞_ + *B*_∞_, hence

$$\frac{u}{x}R_{\infty }-\frac{Q}{y}=M-S_{\infty }-R_{\infty }.$$

Solving for *R*_∞_,

$$R_{\infty }=\frac{x}{x+u}(M-S_{\infty }+\frac{Q}{y}),$$

and substituting this into Eq (11), we obtain

$$B_{\infty }=\frac{(M-S_{\infty })uy-Qx}{y(u+x)}.$$ (12)

Now we go back to Eq (6), divide by *u* and use the relation (12) to get

$$\begin{array}{ll} \text{ln}\;S_{T} & +\;\frac{y}{uN_{T}}D_{T}+\frac{v}{uN_{T}}H_{T}+\left(\frac{\tilde{\varphi }}{\tilde{b}N_{T}}+\frac{y}{uN_{T}}\right)B_{T}\\ & =\;\text{ln}\;S_{\infty }+\left(\frac{\tilde{\varphi }}{\tilde{b}N_{T}}+\frac{y}{uN_{T}}\right)\frac{\left(M-S_{\infty }\right)uy-Qx}{y\left(u+x\right)}. \end{array}$$

Using Eq (4), we find

$$\begin{array}{ll} \text{ln}\;S_{T} & +\;\frac{y}{uN_{T}}D_{T}+\frac{v}{uN_{T}}H_{T}+\left(\frac{\tilde{\varphi }}{\tilde{b}N_{T}}+\frac{y}{uN_{T}}\right)B_{T}\\ & =\;\text{ln}\;S_{\infty }+\frac{{𝓡}_{c}}{S_{T}}\left(M-S_{\infty }-\frac{Qx}{yu}\right). \end{array}$$ (13)

Substitute *Q* from Eq (9) into Eq (13) and obtain

$$\begin{array}{ll} \text{ln}\;S_{T} & +\;\frac{y}{uN_{T}}D_{T}+\frac{v}{uN_{T}}H_{T}+\left(\frac{\tilde{\varphi }}{\tilde{b}N_{T}}+\frac{y}{uN_{T}}\right)B_{T}\\ & =\;\text{ln}\;S_{\infty }+\frac{{𝓡}_{c}}{S_{T}}\left(M-S_{\infty }\right)-\frac{{𝓡}_{c}}{S_{T}}\left(R_{T}+\left(\frac{z}{y}-\frac{xv}{yu}\right)H_{T}-\frac{x}{u}D_{T}-\frac{x}{u}B_{T}\right). \end{array}$$

We use the definition (10) and reorganize the last relation in a more convenient form:

$$\begin{array}{ll} \text{ln}\;S_{T}-\text{ln}\;S_{\infty }= & \frac{{𝓡}_{c}}{S_{T}}\left(N_{T}-S_{\infty }\right)-\frac{{𝓡}_{c}}{S_{T}}R_{T}\\ & +D_{T}\underset{=\frac{\tilde{\varphi }}{\tilde{b}N_{T}}}{\underset{︸}{\left(\frac{{𝓡}_{c}}{S_{T}}\left(1+\frac{x}{u}\right)-\frac{y}{uN_{T}}\right)}}\\ & +B_{T}\underset{=0}{\underset{︸}{\left(\frac{{𝓡}_{c}}{S_{T}}\left(1+\frac{x}{u}\right)-\frac{y}{uN_{T}}-\frac{\tilde{\varphi }}{\tilde{b}N_{T}}\right)}}\\ & +H_{T}\left(\frac{{𝓡}_{c}}{S_{T}}\left(1-\frac{z}{y}+\frac{xv}{yu}\right)-\frac{v}{uN_{T}}\right). \end{array}$$

All in all we have

$$\begin{array}{ll} \text{ln}\;S_{T}-\text{ln}\;S_{\infty }\;= & \frac{{𝓡}_{c}}{S_{T}}\left(N_{T}-S_{\infty }\right)-\frac{{𝓡}_{c}}{S_{T}}R_{T}\\ & +\frac{\tilde{\varphi }}{\tilde{b}N_{T}}D_{T}+H_{T}\left(\frac{{𝓡}_{c}}{S_{T}}\left(1-\frac{z}{y}+\frac{xv}{yu}\right)-\frac{v}{uN_{T}}\right). \end{array}$$

Note that in the special case *T* = 0, we may assume *S*_0_ ≈ *N*_0_, *D*_0_ ≈ *H*_0_ ≈ *R*_0_ ≈ 0, and we retain the standard final size relation

$$\ln(\frac{S_{0}}{S_{\infty }})={𝓡}_{0}(1-\frac{S_{\infty }}{S_{0}}),$$

where the index zero indicates the initial value of the variable.
